# 611 Progressing necrosis, how to make it stop? Clostridial collagenase debridement salvages the zone of stasis

**DOI:** 10.1093/jbcr/irac012.239

**Published:** 2022-03-23

**Authors:** Rajiv Sood, Robert N Bearden, Rosie Frederick

**Affiliations:** JMS/BRCA, Augusta, Georgia; Smith and Nephew, Fort Worth, Texas; Smith+Nephew, Fort Worth, Texas

## Abstract

**Introduction:**

In burns, conversion of partial thickness to deeper, full-thickness burns is largely attributed accumulation of chemical and mechanical mediators altering physiologic processes of tissue viability. Studies suggest that c. collagenase debridement digest denatured collagen without degrading otherwise intact collagen in granulation and healthy tissue. Moreover, modulating cellular responses to foster an anti-inflammatory milieu. To better understand c. collagenase effects in treatment of burns and salvaging the zone of stasis, we evaluated its ability to effectively debride while minimizing secondary necrosis for translative understanding associated with our clinical algorithm

**Methods:**

In this prospective, controlled animal study, deep-partial thickness burns were created on the caudal dorsa. Following burn injuries, wounds were immediately cleaned and treated with either c. collagenase or a hydrogel. Daily treatment was provided. At selected time points, punch biopsies were obtained from unburned interspaces, fixed and prepared for evaluation. In tandem with pathological and histological assessment, samples were examined for cellular and analyte constituents

**Results:**

Gross images and histology revealed, c. collagenase treatment reduced necrosis and apoptosis. In control burns, papillary and reticular dermal collagen was shown to be disorganized and advancing in necrosis as exhibited by significant (p< 0.001) staining of HMGB1. In contrast, the interstices of c. collagenase treated burns demonstrated earlier sloughing of the epidermal layer with a clearly defined basement membrane and significantly (p< 0.001) reduced HMGB1 staining. Moreover, activated caspase 3a staining was significantly (p< 0.05) reduced in c. collagenase treated burn wounds. In assessing the inflammatory response, c. collagenase-treated wounds exhibited significantly (p< 0.05) greater neutrophil influx at day 1, with macrophage recruitment throughout days 1, 2, and 4. In further evaluation, macrophage polarization to MHC II and vascular network maintenance was significantly (p< 0.05) increased in c. collagenase treated wounds

**Conclusions:**

These data corroborate our clinical treatment algorithm, supported by presented case studies. Providing further evidence to potential cellular mechanisms to complement the clinical scenario. Thus, warranting inclusion of early treatment of c. collagenase debridement to preserve tissue from progressive necrosis

